# Onychophagia Induced Melanonychia, Splinter Hemorrhages, Leukonychia, and Pterygium Inversum Unguis Concurrently

**DOI:** 10.1155/2018/3230582

**Published:** 2018-01-30

**Authors:** Sezin Fıçıcıoğlu, Selma Korkmaz

**Affiliations:** ^1^Department of Dermatology, Trakya University Faculty of Medicine, Balkan Yerleskesi, Edirne, Turkey; ^2^Department of Dermatology, Suleyman Demirel University Faculty of Medicine, Isparta, Turkey

## Abstract

Onychophagia, which refers to compulsive nail-biting behavior, is common among children and young adults. Onychophagia can cause destruction to the cuticle and nail plate, leading to shortening of nails, chronic paronychia, and secondary infections. Relatively uncommon effects include pigmentary changes, such as longitudinal melanonychia and splinter hemorrhages. We report a case of a young adult with longitudinal melanonychia, splinter hemorrhages, punctate leukonychia, and pterygium inversum unguis, concurrently induced by onychophagia. Importantly, patients usually do not report this behavior when asked about nail-related changes. Even upon questioning, they may deny nail-biting behavior. As in many other dermatological disorders, dermoscopy can be helpful in the diagnosis of nail disorders.

## 1. Introduction

Onychophagia, which refers to compulsive nail-biting behavior, is common among children and young adults. It seems to be related to obsessive-compulsive spectrum disorder and usually cooccurs with psychopathological symptoms [1, 2]. Onychophagia can cause numerous nail changes, including chronic paronychia, longitudinal melanonychia, splinter hemorrhages, nail dystrophy, and partial or total loss of nails [2, 3].

Either melanocytic activation or melanocytic proliferation can be responsible for melanonychia. Melanocytic proliferation is associated with brown-blackish pigmentation, whereas the pigmentation is lighter or even grayish in the presence of melanocytic activation [4]. Melanocytic activation is the most common cause of benign melanonychia in adults. Repeated trauma, periungual tumors, drugs, or systemic diseases can induce melanocytic activation. The activation of melanocytes can also be idiopathic, such as in ethnic-type nail pigmentation, lentigo of the nail apparatus, and Laugier–Hunziker syndrome [3, 5–7].

Splinter hemorrhages are the result of damage to the delicate spiral arteries of the nail bed [8]. The etiology of splinter hemorrhages includes dermatoses (e.g., psoriasis and lichen planus), connective tissue diseases, vasculitis, drugs, particularly kinase inhibitors, infectious diseases, such as acute endocarditis and meningococcemia, and renal failure [4, 8]. In addition, splinter hemorrhages may be idiopathic, as seen in elderly, or they can be caused by various types of trauma, such as playing percussion instruments, housework, sports, and habits/tics.

Leukonychia arising from nail plate abnormalities is called the true form, and it can be longitudinal, transverse, or punctate. In true leukonychia, the discoloration will not disappear when pressure is applied to the nail plate. In contrast, in apparent leukonychia, where the problem is in the nail bed, the discoloration will disappear when pressure is applied [9].

## 2. Case Report

**Figure 1:**
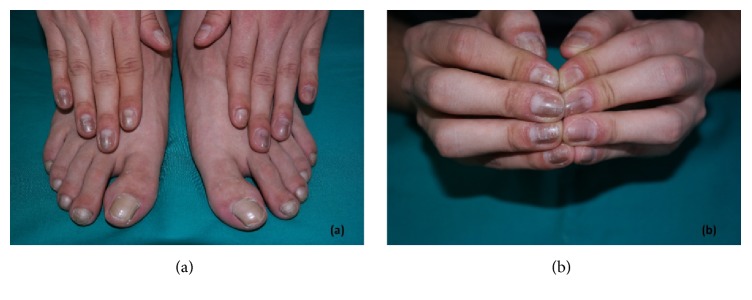
Whole fingernails and toenails of our patient. Normal toenails except for the second ones which have transverse lamellar splitting (a) and grayish pigmented bands with varying widths and intensities on fingernails, predominantly on right hand; loss of cuticle and erythema of proximal nail folds which can also be seen on both hands (b).

**Figure 2:**
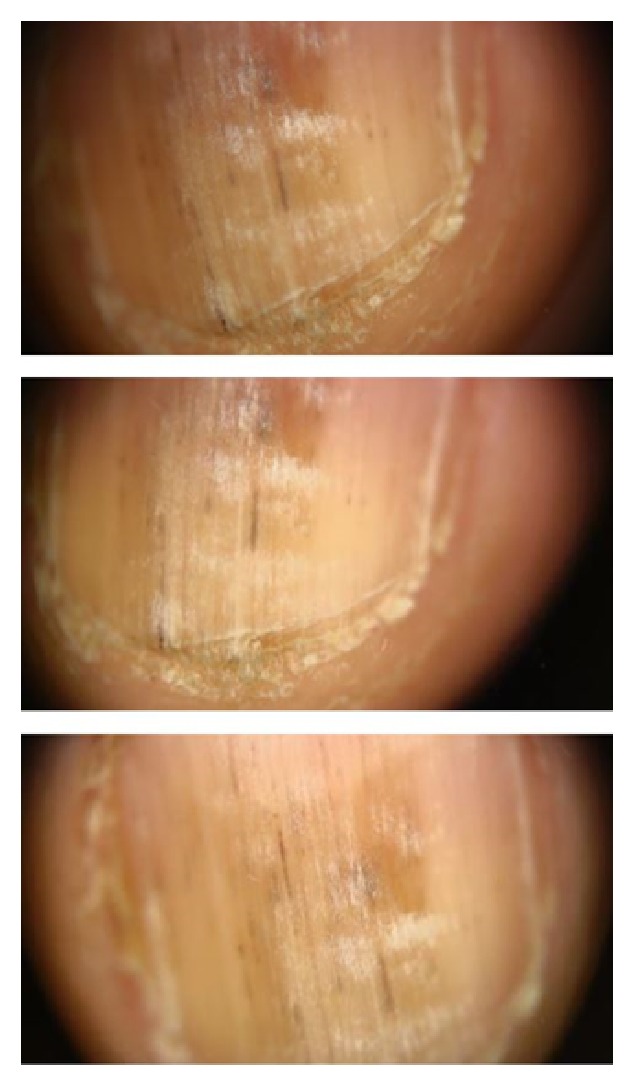
Dermoscopic evaluation of right second nail yields longitudinal gray regular lines on a grayish background, splinter hemorrhages as blackish linear streaks, and true leukonychia as it did not disappear with pressure and hyperkeratosis in hyponychium.

A 20-year-old male presented with grayish pigmentation on multiple fingernails for a duration of more than two years. He was otherwise healthy, and a physical examination revealed longitudinal hyperpigmented bands of varying widths on the middle parts of his fingernails especially on the right hand. His toenails were normal, except for the second toes of both sides. These showed evidence of transverse lamellar splitting, which was thought to be the result of repeated traumas from ill-fitting footwear. His fingernails also showed punctate leukonychia; small, blackish linear streaks between hyperpigmented bands; and hyperkeratosis in the hyponychium, which obliterated the distal groove, again predominantly on the right hand (Figure 1, informed consent has been obtained from the patient). In addition, the proximal nail folds of all the fingers were swollen, with erythematous patches, and no cuticles were visible on any of the fingernails. The results of laboratory investigation, including a hemogram (white blood cell count: 7430/*μ*L, normal range: 4230–9070/*μ*L, hemoglobin: 15.7 gr/dl, normal range: 13.7–17.5 gr/dl, and hematocrit: 45.6%, normal range: 40.1–51%), liver, renal, and thyroid function tests, and iron status, were normal. Only vitamin B12 (210 pg/ml, normal range: 211–911 pg/ml) and folic acid (4.29 ng/mL, normal range: 5.38–24 ng/mL) levels were slightly decreased. Immunological studies for antinuclear antibodies were negative. Chest and wrist radiography for the investigation of sarcoidosis were normal. There was no known history of psoriasis or lichen planus and dermatological examination did not reveal any suspicious lesion for them. In addition, a mycological examination with potassium hydroxide smear and a fungal culture was negative. There was no remarkable medical history or contact history of detergents or special chemicals. Family history for similar nail findings or connective tissue diseases was also negative. A dermoscopic examination revealed faintly pigmented longitudinal bands on the middle parts of the patient's fingernails with various intensities, being predominant on the right side. The pigmentation did not extend into the proximal nail fold. The leukonychia did not disappear during the examination when pressure was applied to the nail plates, and they were laid out on the pigmented bands. The blackish linear streaks observed earlier in the physical examination were splinter hemorrhages. In addition, the dermoscopic examination clearly revealed hyperkeratosis in the hyponychium and adherence of the nail bed to the nail plate, raising the suspicion of pterygium inversum unguis (Figure 2). However, it was difficult to identify subungual extension of the hyponychium because the patient's nails were short. Upon questioning about pain while clipping his nails related to the hyponychium hyperkeratosis, the patient admitted that he had been biting his nails for nearly 4 years, becoming more intense in the last two years.

## 3. Discussion

The repeated trauma in onychophagia induces melanocytic activation and leads to melanonychia which extends by longitudinal spreading from the nail bed to the distal end of the nail plate. This longitudinal melanonychia is grayish in color, with regular gray parallel lines on a gray background [3, 6]. Onychophagia-induced melanonychia can accompany other trauma-induced nail changes, such as distal nail plate splitting, onychoschizia, and nail plate hypertrophy [3]. Our case had grayish longitudinal parallel lines on nearly all nail plates and they were accompanied with chronic paronychia and onychoschizia.

Splinter hemorrhages in our case were mostly black and located distally so we thought that they were related to onychophagia. This is because when they are traumatic, they are mostly black and located distally, whereas they are red and proximal in the presence of systemic diseases [8].

Multiple punctate leukonychia seen in our case was true leukonychia as it did not disappear upon pressure. True leukonychia can be seen in inherited syndromes, alopecia areata and proximal subungual onychomycosis, or acquired as a result of severe illness or chemotherapeutic exposure [9]. It can also be idiopathic. Trauma can cause true leukonychia [2, 9]. In the present case, we attributed the punctate leukonychia to onychophagia, as they were mostly visible on the pigmented middle parts, where trauma can affect the nail plate.

Pterygium inversum unguis is an infrequent disorder, where an exaggerated stratum corneum in the distal nail bed or hyponychium leads to obliteration of the distal groove. It can be congenital or acquired, and acquired forms have been reported frequently in connective tissue diseases, such as systemic sclerosis [10]. We propose that pterygium inversum unguis may also be associated with reactive hyperkeratosis and an inflammatory reaction to repeated trauma, as described in the present case.

In conclusion, in the present case, all the nail changes were attributed to onychophagia as the shared etiology was trauma. In the literature, we found no case reports of longitudinal melanonychia, splinter hemorrhages, punctate leukonychia, and pterygium inversum unguis appearing concurrently with onychophagia. In the present case, the multiple nail changes may have been due to the severe nature of the trauma and the long duration of onychophagia in a young adult. It is important to question patients specifically about nail-biting or picking behaviors when evaluating nail disorders, as they may not readily admit such behaviors unless questioned. The present case also illustrates the importance of dermoscopes, which are becoming indispensable for dermatologists, in diagnosing diseases in all kinds of patient populations.
